# Isoform-Level Transcriptome Analysis of Peripheral Blood Mononuclear Cells from Breast Cancer Patients Identifies a Disease-Associated *RASGEF1A* Isoform

**DOI:** 10.3390/cancers16183171

**Published:** 2024-09-16

**Authors:** Helena Čelešnik, Mario Gorenjak, Martina Krušič, Bojana Crnobrnja, Monika Sobočan, Iztok Takač, Darja Arko, Uroš Potočnik

**Affiliations:** 1 Faculty of Chemistry and Chemical Engineering, University of Maribor, Smetanova ulica 17, 2000 Maribor, Slovenia; helena.celesnik@um.si; 2 Faculty of Medicine, University of Maribor, Taborska ulica 8, 2000 Maribor, Slovenia; mario.gorenjak@um.si (M.G.); martina.krusic1@um.si (M.K.); monika.sobocan3@um.si (M.S.); iztok.takac@um.si (I.T.); darja.arko@um.si (D.A.); 3 Division of Gynecology and Perinatology, University Medical Center Maribor, Ljubljanska ulica 5, 2000 Maribor, Slovenia; bojana.crnobrnja@ukc-mb.si; 4 Department for Science and Research, University Medical Centre Maribor, Ljubljanska ulica 5, 2000 Maribor, Slovenia

**Keywords:** breast cancer, peripheral blood, isoform-level RNA-seq, *RASGEF1A ENST00000374459*, ctDNA, Ki-67

## Abstract

**Simple Summary:**

Peripheral blood analyses can offer a minimally invasive view into systemic immunity during cancer and can lead to the identification of biomarkers for cancer screening and therapeutic management. While a limited number of studies have reported blood transcriptome in breast cancer (BC) using RNA-seq analysis, our study is the first that aimed to identify potential BC biomarkers by analyzing transcriptome at an isoform level in peripheral blood mononuclear cells (PBMCs) from BC patients and healthy women. Our approach has led to the identification of an isoform of the *RASGEF1A* gene, the *ENST00000374459* transcriptional variant, as a promising blood mRNA biomarker for distinguishing BC and healthy subjects. Additionally, our association analysis with clinicopathological characteristics revealed that lower *ENST00000374459* expression in PBMCs of breast cancer patients was associated with higher proliferation and circulating tumor DNA (ctDNA) shedding, thereby linking expression of this isoform in blood immune cells to cancer progression and spreading.

**Abstract:**

**Background:** Breast cancer (BC) comprises multiple subtypes with distinct molecular features, which differ in their interplay with host immunity, prognosis, and treatment. Non-invasive blood analyses can provide valuable insights into systemic immunity during cancer. The aim of this study was to analyze the expression of transcriptional isoforms in peripheral blood mononuclear cells (PBMCs) from BC patients and healthy women to identify potential BC immune biomarkers. **Methods:** RNA sequencing and isoform-level bioinformatics were performed on PBMCs from 12 triple-negative and 13 luminal A patients. Isoform expression validation by qRT-PCR and clinicopathological correlations were performed in a larger cohort (156 BC patients and 32 healthy women). **Results:** Transcriptional analyses showed a significant (*p* < 0.001) decrease in the *ENST00000374459 RASGEF1A* isoform in PBMCs of BC compared to healthy subjects, indicating disease-related expression changes. The decrease was associated with higher ctDNA and Ki-67 values. **Conclusions:** The levels of the *RASGEF1A* transcriptional isoform *ENST00000374459* may have the potential to distinguish between BC and healthy subjects. The downregulation of *ENST00000374459* in breast cancer is associated with higher proliferation and ctDNA shedding. Specialized bioinformatics analyses such as isoform analyses hold significant promise in the detection of biomarkers, since standard RNA sequencing analyses may overlook specific transcriptional changes that may be disease-associated and biologically important.

## 1. Introduction

Breast cancer (BC) is among the leading health concerns among women worldwide due to its high prevalence and mortality [1]. This heterogenous cancer comprises multiple subtypes that differ in prognosis and require different therapeutic regimens. The clinical subtypes are based on the expression of the estrogen receptor (ER) and the progesterone receptor (PR) and the amplification of the HER2 (ERBB2) receptor and include *hormone receptor (HR)-positive* (HR+/HER2−, also ER+/PR+/HER2−), *triple-positive* (HR+/HER2+, also ER+/PR+/HER2+), *HER2-positive* (ER−/PR−/HER2+, also HER2+), and *triple-negative breast cancer* (TNBC) (HR−/HER2−, also ER−/PR−/HER2−) [2,3,4]. In addition, the molecular expression profiling of BC tissues has led to the classification of several *intrinsic BC subtypes* with different prognoses and survival: *Luminal A* (ER+ and/or PR+, HER2−, proliferation marker Ki-67 < 14%), *Luminal B* (ER+ and/or PR+, HER2−, Ki-67 ≥ 14%; or ER+ and/or PR+, HER2+, any Ki-67), *HER2(+)/HER2-enriched* (ER-, PR-, HER2+, any Ki-67), and *basal-like (triple-negative)* (ER−, PR−, HER2−, any Ki-67) [4,5]. Intrinsic subtypes overlap with clinical subtypes to a great extent, though not completely [6].

Despite significant progress in tissue-based classifications, a substantial gap remains in understanding how blood immune-related biomarkers, such as transcriptome changes in peripheral blood, can be used to distinguish between BC subtypes. Additionally, while transcriptional isoforms have shown potential as powerful cancer biomarkers, their specific role in peripheral blood in BC patients is still largely unexplored, as further discussed below.

Studies of peripheral blood can provide valuable insights into the interplay between the host systemic immunity and cancer [7,8]. This may aid in identifying biomarkers related to immune responses in BC, enhancing our understanding of disease progression and supporting advancements in cancer screening, subtype characterization, diagnosis, prognosis, and therapy selection. Several research groups have reported that mRNA biomarkers from peripheral blood can be used as “surrogate” biomarkers for various solid tumors [9,10,11,12]. However, while transcript abundance changes in peripheral blood cells are very important in BC, basic gene expression analyses may not be able to fully explain the phenotypes observed during cancer development. The complex eukaryotic gene expression involves the generation of various coding mRNA variants or isoforms from single genes through mechanisms that include selection of transcription start sites, alternate UTR usage, intron retention, alternatively spliced exons, etc. [13,14,15]. Importantly, aberrant use of one isoform over another is frequently associated with cancer [14,16,17,18]. For instance, a specific 3′UTR variant of *HNRNPA1* is downregulated in BC tissues in favor of a more stable isoform, from which more HNRNPA1 protein is produced. Importantly, this correlates with poorer survival [19]. Specific genes important in BC, including *BRCA1*, *TP53*, *PTEN*, and *CD44*, exhibit cancer-specific splice isoforms. Moreover, alternative splicing of *CD44* and other genes in BC cell lines has been shown to play a role in epithelial-to-mesenchymal transition, a process associated with metastasis [20,21,22,23]. 

Notably, BC tumor tissues of individual cancer subtypes display distinct gene expression profiles, including specific transcriptional isoforms [14,24]. RNA sequencing of ER-positive and triple-negative BC tumors has identified specific sets of isoforms that differentiated these subtypes with even higher fidelity than standard mRNA expression profiles [14]. Additionally, dysregulation of splicing in BC subtypes has been shown to be influenced by specific RNA processing factors, since several RNA processing factors were differentially expressed between tumor subtypes and/or regulated by estrogen receptor [14].

Limited studies examining transcriptional isoforms in the peripheral blood cells of BC patients have revealed alterations in the splicing of *BRCA1/2* and *BRCA1-associated RING domain (BARD1)* as significant disease-causing mechanisms [21,25,26]. Furthermore, high expression of the REST-N50 splice variant was observed in nucleated blood cells of locally advanced BC patients. Neoadjuvant therapy led to a decrease in REST-N50 levels, indicating a potential of this variant for the evaluation of therapy effectiveness [27]. Some alternatively spliced mRNA variants (ASVs) identified in the primary tumor have also been reported in the peripheral blood of cancer patients [27]. While isoform investigations in BC tissues have identified BC subtype-specific changes, there has been no study of transcriptional isoforms in blood cells that could specifically distinguish individual BC subtypes.

Together, the above-mentioned findings indicate that transcriptional isoforms have a promising potential to serve as cancer biomarkers [28]. A detailed characterization of the BC-associated mRNA variant repertoire may uncover new oncogenic mechanisms and host immune responses, which may in turn be relevant for the development of therapeutic strategies. Given the promising potential of transcriptional isoforms as cancer biomarkers, this study aimed to bridge the gap by characterizing mRNA isoform expression in peripheral blood mononuclear cells (PBMCs) from BC patients and healthy controls. Moreover, two BC subtypes (TNBC and luminal A), which are generally considered to differ significantly in immunogenicity [29,30], were investigated using RNA-seq to identify the underlying mechanisms leading to differences in systemic immunity between BC subtypes. While TNBC is known for its high immunogenicity due to its genomic instability and a high level of tumor-infiltrating lymphocytes [29,31], luminal A is considered less immunogenic [29,32]. Our RNA-seq analysis represents the first isoform-level transcriptome analysis in peripheral blood of BC patients.

## 2. Materials and Methods

### 2.1. The Study Population and Clinicopathological Characteristics

The pilot (discovery) cohort for RNA-seq comprised 12 TNBC and 13 luminal A female BC patients. The validation cohort for qRT-PCR analyses comprised 156 female BC patients (including the 26 patients from the discovery cohort) and 32 healthy females. All cancer patients had histologically confirmed BC and were treated at the University Medical Centre Maribor (UMC), Slovenia, where the clinicopathological characteristics (ER, PR, HER-2 status, histological type, localization, grade, tumor size, lymph node status, Ki-67) (Table 1) were determined by experienced BC pathologists according to the standard procedures of the pathology laboratory. The healthy controls were enrolled at the Faculty of Medicine at the University of Maribor. All participants provided written informed consent for their participation in this study. This study was approved by the UMC Maribor Medical Ethics Committee (reference number UKC-MB-KME-09/19) and was carried out according to the principles of the Declaration of Helsinki. The blood samples were acquired from BC patients prior to treatment.

### 2.2. Processing of Blood Samples

Whole blood (12 mL) was collected into BD Vacutainer EDTA vials (Becton Dickinson, Franklin Lakes, NJ, USA) and processed immediately after drawing. The samples were centrifuged (300× *g*, 20 min) to collect the plasma. The remainders of the samples were subjected to gradient centrifugation using Lympholyte-H (CL5020; Cedarlane, Burlington, ON, Canada) according to the manufacturer’s instructions to obtain PBMCs for expression analyses.

Circulating DNA was isolated from plasma by QIAamp Circulating nucleic acid kit (Qiagen, Hilden, Germany). The genome-wide Z score (Table 1) for estimation of ctDNA content in plasma was determined by the mFAST-SeqS method as previously described [33]. RNA was prepared from PBMCs using the innuPREP RNA Mini Kit (Analytik Jena, Jena, Germany) according to the manufacturer’s protocol.

### 2.3. Sequencing of RNA Isolated from PBMCs

RNA-seq was performed on 25 RNA samples extracted from PMBCs. Pair-end RNA-seq (oligo dT, stranded mRNA library, DNBseq platform, PE150, 20 M reads/6 Gb clean data) was performed at BGI (Hong Kong, China) using a NEBNext^®^ Poly(A) mRNA Magnetic Isolation Module and BGI kit.

### 2.4. RNA-Seq Data Alignment and Identification of Differentially Expressed Isoforms

Raw .fastq files were first assessed for quality using FastQC software (0.11.9, Babraham Bioinformatics, Cambridge, UK) [https://www.bioinformatics.babraham.ac.uk/projects/fastqc/; accessed on 13 September 2024]. Subsequently, trimming of technical sequences was performed using the Trimmomatic tool (version 0.39, USADEL LAB, Aachen, Germany) [34]. Transcript-specific RNA-seq analysis was performed using the Kallisto pseudoalignment program (Pachter Lab, Berkeley, CA, USA) with the GRCh37 reference genome [35]. Furthermore, the R 4.2.1 environment (R Core Team 2020, Vienna, Austria) and a pipeline described elsewhere [36] were used for further processing of estimated counts and tpms. Statistically significant differential gene expression was identified using a false discovery rate (FDR) ≤ 0.1 and log2 count per million (logCPM) ≥ 1.

### 2.5. Preparation of cDNA and Isoform-Specific RT-qPCR Analysis

Total RNA (200 ng) was used to prepare cDNA by using the High-Capacity cDNA Reverse Transcription Kit (Applied Biosystems, Foster City, CA, USA) following the manufacturer’s instructions. Quantitative reverse transcription PCR (RT-qPCR) was performed on the LightCycler^®^ 480 System (Roche, Basel, Switzerland) using the cDNA samples and the LightCycler^®^480 SYBRGreen I Master reaction mix (Roche, Basel, Switzerland). To amplify different *RASGEF1A* transcriptional variants, *374459*-specific and *395810*-specific forward primers and a common exon junction-spanning reverse primer were used. The optimal PCR conditions and efficiency were assessed for each primer pair. The primer sequences, final primer concentrations, and annealing temperatures were as follows: for *ENST00000374459* (5′-CCGGCGGCCAGAATGTTCCTGGA-3′ and 5′-TACGTCCTATCGGGGTAATAGTCCACC-3′, 400 nmol, 52 °C); for *ENST00000395810* (5′-AGCGACGCTGGCCCGGACCG-3′ and 5′-TACGTCCTATCGGGGTAATAGTCCACC-3′, 400 nmol, 62 °C). Target gene expression levels were calculated relative to the endogenous control *18S* rRNA (5′-GCAATTATTCCCCATGAACG-3′ and 5′-GGGACTTAATCAACGCAAGC-3′) and described as relative expression levels using the 2^−ΔΔCT^ method [37] for 374459 and the Pfaffl method [38] for *395810*. Ct values > 40 were considered negative. 

Statistical analyses of isoform expression were performed with SPSS Statistics 28 (IBM Corporation, Armonk, NY, USA) using the Mann–Whitney U test. The results were defined as significant when *p* < 0.05.

## 3. Results

### 3.1. RNA Sequencing Identified Differences in Isoform Expression between Luminal A and TNBC Patients

**Figure 1:**
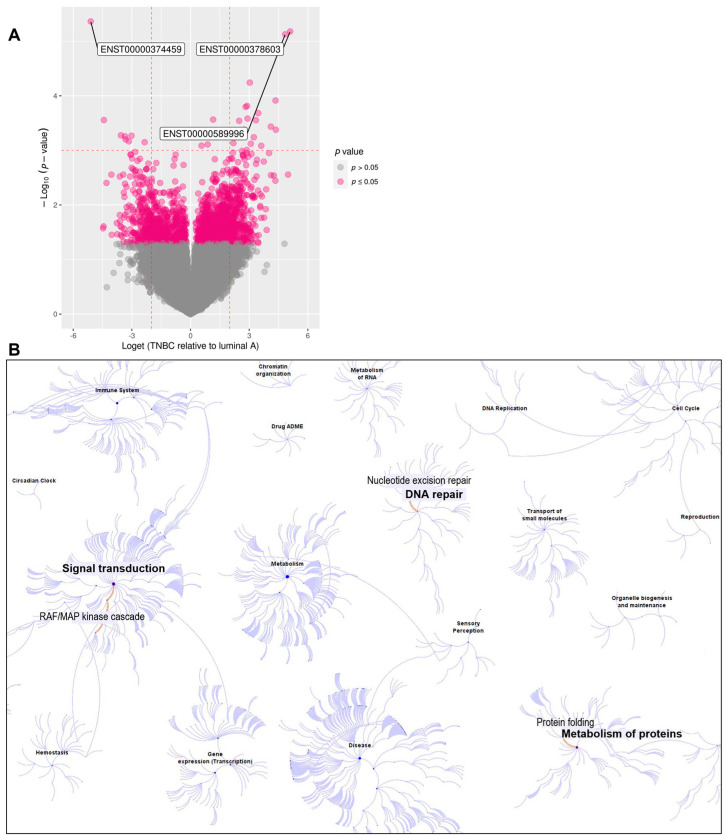
(**A**) Volcano plot showing differentially expressed isoforms (DEIs) between 13 luminal A and 12 triple−negative treatment−naïve BC patients. The top DEIs were the *ENST00000374459* transcriptional variant of the *RASGEF1A* gene, which had lower PBMC expression in TNBC than luminal A; the *ENST00000589996* transcriptional variant of the *TBCB* gene (with higher expression in TNBC); and the *ENST00000378603* transcriptional variant of the *DDB2* gene (with higher expression in TNBC). (**B**) Overview of Reactome pathways [39], highlighting the signal transduction (RAF/MAP kinase cascade) pathway involving *RASGEF1A*, the DNA repair (nucleotide excision repair) pathway involving *DDB2*, and the metabolism of proteins (protein folding) pathway involving *TBCB*.

High-throughput RNA sequencing was performed using mRNA isolated from PBMCs of 13 luminal A and 12 triple-negative treatment-naïve BC patients in order to identify statistically significant differentially expressed isoforms (DEIs). The sequencing data were analyzed at the transcript isoform level by using the R 4.2.1 environment (R Core Team 2020, Vienna, Austria). The analysis rendered three transcriptional variants belonging to three different genes as significantly differentially expressed between TNBC and luminal A: the *Ras-GEF Domain-Containing Family Member 1A (RASGEF1A) ENST00000374459* variant, the *Tubulin Folding Cofactor B (TBCB) ENST00000589996* variant, and *the Damage-Specific DNA Binding Protein 2 (DDB2) ENST00000378603* variant (Figure 1A and Table S1). 

Function and pathway analyses were performed to gain insight into the biological context of these results. Analyses using the Reactome pathway database [39] (Figure 1B), UniProt [40] (Table 2), and the STRING database [41] (Figure S1) revealed that the three significantly differentially expressed genes do not share the same or similar pathways but are instead involved in distinct, non-overlapping cellular pathways. *RASGEF1A* participates in the Ras/Raf/MAP kinase signaling cascade, with gene ontology (GO) annotations related to this gene (Table 2) including guanyl-nucleotide exchange factor activity. *DDB2* encodes a protein that participates in nucleotide excision repair, with GO annotations related to DDB2 including damaged DNA binding. *TBCB* encodes a protein involved in the regulation of tubulin heterodimer dissociation, with GO annotations related to TBCB including alpha-tubulin binding. Similarly, the Reactome analysis identified these genes in the signal transduction pathway (*RASGEF1A*), the DNA repair pathway (*DDB2*), and the protein folding pathway (*TBCB*) (Figure 1B), while the STRING analysis identified the RASGEF1A, DDB2, and TBCB protein interactors in these pathways (Figure S1).

In the continuation of our research, we focused on *RASGEF1A* due to its distinct association with the RAS pathway, a crucial signaling cascade implicated in cancer development and progression, with dysregulation of the Ras signaling pathway reported to lead to uncontrolled cell proliferation and resistance to apoptosis [42]. Furthermore, its close homolog, *RASGEF1B*, has been reported to play a functional role in macrophages and chemotaxis [43,44,45], which hinted at the possibility that RASGEF1 proteins assume multifaceted roles in both cancer-related signaling and immune modulation. 

**Figure 2:**
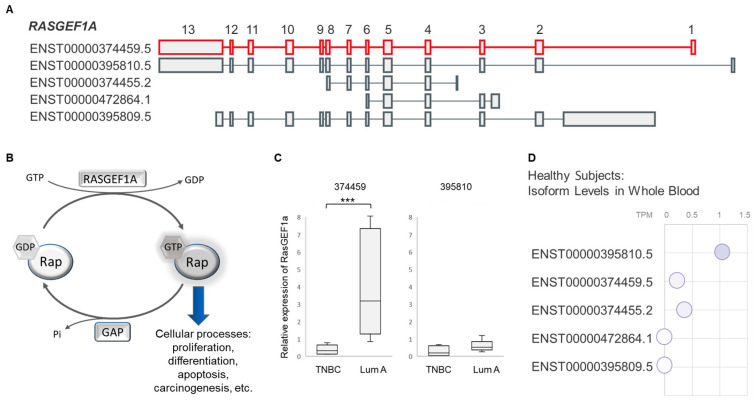
RNA-seq analysis of PBMCs from 12 TNBC and 13 luminal A female BC patients identified changes in expression of the *RASGEF1A 374459* variant. (**A**) *RASGEF1A* variants. The isoform *374459* is depicted in red with exons numbered 1–13; adapted from the GTEx portal [48]; (**B**) RASGEF1A belongs to the GEF (guanine nucleotide exchange factor) family of proteins, which mediate GDP release and GTP binding to the Rap proteins, thereby activating them. Inactivation of the Rap proteins is initiated by their intrinsic GTPase activity, which is enhanced by the GTPase activating (GAP) proteins. The Rap proteins belong to the Ras family, whose members are known to participate in signaling pathways that control a diverse array of cellular processes (i.e., cell proliferation, differentiation, etc.); (**C**) the patient PBMC samples from RNA-seq analysis (i.e., the pilot cohort) were subjected to qRT-PCR validation with *374459*-specific (left) and *395810*-specific (right) primers; Mann–Whitney U test (*** *p* < 0.001). (**D**) The levels of *RASGEF1A* isoforms in whole blood of healthy subjects (from RNA-seq data) (adapted from the GTEx portal) [48].

While five different isoforms have been reported for *RASGEF1A* (Figure 2A), RNA-seq returned only the *ENST00000374459* variant (hereinafter *374459*) as significantly downregulated in peripheral immune cells of TNBC patients. RASGEF1A is a member of the GEF (guanine nucleotide exchange factor) family of proteins, which mediate GDP release and GTP binding to the Rap proteins (Figure 2B). The *Rap* genes belong to the *Ras* family, known to be frequently mutated in cancer [46,47]. Considering that GEFs activate their targets through GTP exchange, not through transcriptional regulation, our RNA-seq analysis expectedly showed no significant differences in expression of the *Rap* genes between TNBC and luminal A.

The PBMC samples from RNA-seq were further subjected to qRT-PCR validation with isoform-specific primers capable of detecting two *RASGEF1A* variants that differ in the first exon and the first intron sequence: the *374459* and the *ENST00000395810* (hereinafter *395810*) isoforms (Figure 2A,C). In line with RNA-seq results, distinctive PBMC isoform expression was observed in validation analysis: while *374459* expression was significantly lower (*p* < 0.001) in TNBC samples, expression of *395810* was similar between the two BC subtypes (Figure 2C).

It may be worth noting that our initial isoform-indiscriminate (all-inclusive) whole-transcriptome RNA-seq analysis of PBMCs did not identify *RASGEF1A* as a differentially expressed gene (DEG) between TNBC and luminal A. This is not surprising considering that *395810* is the most represented *RASGEF1A* transcriptional variant in human whole blood, as seen in the publicly available GTEx expression data (Figure 2D), while *374459* is substantially less abundant. Taking into account the homogeneous *395810* expression among BC patient subtypes (Figure 2C), and the low overall presence of *374459* (Figure 2D), any potential expression changes for the less abundant *374459* transcriptional variant can easily be missed by the classical whole-transcriptome RNA-seq analysis, even though these alterations may be biologically important. However, the isoform-level RNA-seq analysis is able to pick up on such changes.

### 3.2. Expression of RASGEF1A Isoforms in a Larger Cohort Comprising BC Patients and Healthy Female Controls

**Figure 3:**
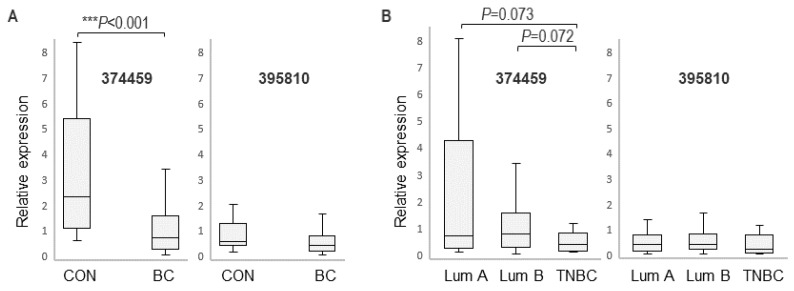
Isoform-specific *RASGEF1A* expression in a larger cohort comprising 32 healthy female subjects (CON) and 156 BC patients (45 luminal A, 90 luminal B, 5 HER2, 16 TNBC). (**A**) qRT-PCR analysis of PBMCs from healthy (CON) and BC subjects (comprising all BC subtypes) with *374459*-specific (left) and *395810*-specific (right) primers; (**B**) qRT-PCR analysis of PBMCs from different BC subtypes with *374459*-specific (left) and *395810*-specific (right) primers (the five HER2-positive samples were not included due to low number). Mann–Whitney U test, *** *p* < 0.001.

Isoform expression was further analyzed in a larger cohort that included 156 female BC patients (average age 60.7 ± 13.4 years) and 32 healthy women (average age 55.6 ± 9.2 years). Quantitative RT-PCR analysis revealed a significantly higher expression of the *374459* variant in PBMCs of healthy controls (*p* < 0.001) compared to BC patients (Figure 3A), while the levels of the *395810* isoform were similar between these groups. This suggested that expression of the *374459* variant is associated with BC and indicated a potential for *374459* as a disease-associated biomarker.

Variant expression was further compared between different subtypes of BC in the larger cohort. Here, the trend of lower *374459* expression in TNBC compared to luminal A persisted; however, it was short of reaching statistical significance (*p* = 0.073) (Figure 3B). A similar situation was observed when *374459* expression was compared between TNBC and luminal B (*p* = 0.072). Hence, despite the significant subtype-specific *374459* expression changes identified by RNA-seq in the pilot cohort, the targeted validation in the larger BC cohort indicated that *374459* expression changes were insufficient to serve for blood-based distinction of BC subtypes. 

Consistent with the pilot cohort, the larger cohort showed similar expression of the *395810* isoform in healthy controls and BC patients as well as within the BC subtypes (Figure 3).

### 3.3. RASGEF1A Isoform Expression and Clinicopathological Characteristics of BC Patients

The expression of *374459* in PBMCs of BC patients was also analyzed with respect to the clinicopathological characteristics (Table 1) of BC patients.

#### 3.3.1. Association with Ki-67 Proliferation Index

**Figure 4:**
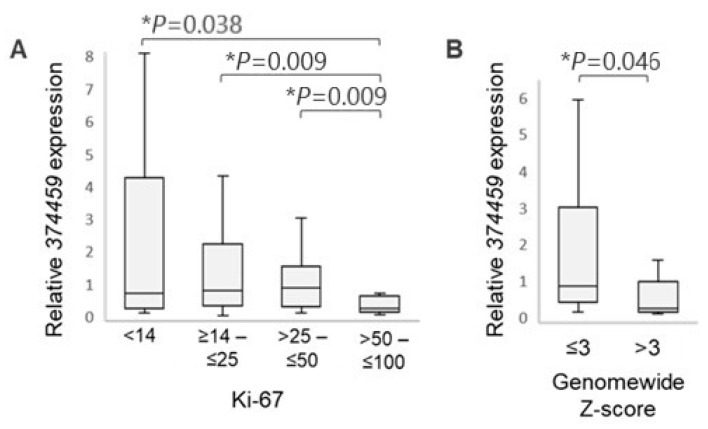
Lower *374459* expression in PBMCs of BC patients showed association with higher Ki-67 proliferation index and ctDNA content. (**A**) qRT-PCR analysis of *374459* expression in PBMCs from BC patients stratified by increasing Ki-67 index; Mann–Whitney U test, * denotes *p* < 0.05. (**B**) qRT-PCR analysis of *374459*-expression in PBMCs from BC subjects with lower (z-score ≤ 3) and higher (z-score > 3) ctDNA content; Mann–Whitney U test, * denotes *p* < 0.05.

The prognostic proliferation marker Ki-67 is routinely evaluated by the clinical pathology labs to assist in BC subtyping and treatment decisions. Nonetheless, there is a lack of consensus regarding the cutoff points for high and low immunohistochemical Ki-67 values; therefore, these tend to vary between laboratories [49]. Taking into account the groupings in published BC studies [50,51,52,53,54], the BC patients in our cohort were stratified into groups with increasing Ki-67 values: group I (Ki-67 < 14%); group II (≥14 to ≤25%); group III (> 25 to ≤50%); and group IV (>50 to ≤100%) (Figure 4A). A significantly lower expression of the *374459* isoform was observed in group IV patients who are characterized by high immunohistochemical Ki-67 staining (>50%) compared to patients with Ki-67 ≤ 50% (Figure 4A), indicating an association between the downregulation of *374459* and tumor proliferation. While there was an incremental decrease in *374459* expression between groups I and II, and between groups II and III, it was not statistically significant (Figure 4A).

#### 3.3.2. Association with Circulating Tumor DNA (ctDNA) Content

Circulating tumor DNAs are small pieces of extracellular DNA released by the dying tumor cells, which contain information on somatic mutations in the tumor cells [55]. We determined the ctDNA content in plasma of BC patients by employing the Modified Fast Aneuploidy Screening Test-Sequencing System (mFAST-SeqS). This method detects tumor-specific aneuploidy in circulating cell-free DNA (cfDNA). The acquired genome-wide mFAST-SeqS z-scores correlate with the tumor content in plasma [33,56,57]. The genome-wide z-scores were determined for 41 BC subjects (Table 1). The patients’ z-scores were <5, except in two subjects who had higher scores. While values below 5 indicate a generally low ctDNA content, we nonetheless observed that patients with z-scores >3 had significantly decreased *374459* expression (*p* = 0.046) compared to those with z-scores ≤3 (Figure 4B), suggesting that the levels of this *RASGEF1A* isoform are inversely correlated with ctDNA amount.

Together, the Ki-67 and ctDNA results indicate that the downregulation of *374459* is associated with tumor proliferation and ctDNA shedding.

#### 3.3.3. Other Clinicopathological Characteristics

When other clinicopathological characteristics were evaluated with regard to *374459* expression, no statistically significant associations were observed (Figure S2). 

## 4. Discussion

Our transcriptional analyses in mononuclear blood cells showed that the *RASGEF1A 374459* transcriptional isoform is significantly downregulated in BC compared to healthy subjects, suggesting disease-associated expression changes, and indicating the ability of *374459* to distinguish between these two groups.

### 4.1. The Advantages of Blood Analyses over Standard Methods for Cancer Detection

We focused on peripheral blood cells because of the important advantages that blood-based cancer indicators offer over tissue markers. For one, collecting peripheral blood is simple, minimally invasive, and cost-efficient [58]. Additionally, evidence indicates that the analysis of blood can enable the detection of very early systemic changes, crucial for cancer screening [59,60,61,62]. In contrast, biopsies are invasive, carry a greater possibility of complications, and may not be suitable for screening purposes. While repeated tissue biopsies can be used to oversee the progression of cancer, single-site biopsies may have selection bias because of tumor heterogeneity and may not provide enough material [13]. On the other hand, peripheral blood is not prone to heterogeneity problems or selection bias and is readily available in sufficient quantities [58]. Analyzing blood may also offer advantages compared to current imaging methods. While mammography is important for screening, it has drawbacks like radiation exposure and physical discomfort. Furthermore, its capacity to identify the tumor in its early stages is hampered by the necessity for the tumor to reach a certain size to be detectable [63]. Also, due to high breast density, around 10% of cancers remain undetected on mammography [64,65].

While our investigation indicated that *374459* may have the capability to distinguish between healthy and breast cancer (BC) subjects, its potential utility as a biomarker needs to be established through replication. In pursuit of validation across different cohorts, we sought to identify an external RNA-seq cohort for further validation. However, we found only two articles reporting peripheral blood RNA-seq analyses in breast cancer, both without their data deposited in public repositories [66,67]. In contrast, available blood transcriptome studies in public repositories such as the GEO Database—NCBI and European Genome-Phenome Archive were performed using array profiling (e.g., GDS3952, GSE27562, GSE47862, EGAD00010001063), rendering them unsuitable for isoform analysis.

### 4.2. RASGEF1A Function

RASGEF1A is a member of the conserved RASGEF1 family of proteins, which includes RASGEF1A, RASGEF1B, and RASGEF1C. This family controls the activity of the Rap protein family (Figure 2B) [68,69]. RASGEF1B has a well-established role in immunity, where it is involved in macrophage signaling, chemotaxis, and cytokine response [43,44,70,71]. On the other hand, there is limited information available about RASGEF1A and RASGEF1C. Specifically, there are no reports yet suggesting a potential role in immunity for RASGEF1A. However, according to PBMC single-cell sequencing data from the Protein Atlas, *RASGEF1A* mRNA has been detected in T-cells, NK cells, and macrophages [72]. 

The Rap proteins (Rap1A, Rap1B, Rap2A, Rap2B, Rap2C) belong to the Ras superfamily and are found in nearly all tissues where they have regulatory roles in growth, differentiation, proliferation, carcinogenesis, cell adhesion, exocytosis, apoptosis, and phagocyte activity [70,71]. Individual Rap members have specific functions. For instance, Rap1 and Rap2 signal through distinct downstream pathways [73]. Moreover, Rap2C is the predominant Rap2 protein in circulating mononuclear leukocytes, but it is not present in platelets [71,73]. 

Interestingly, RASGEF1A and RASGEF1B proteins are highly specific guanine nucleotide exchange factors (GEFs) for Rap2 and do not act on Rap1 or other Ras members [47]. The Rap2 group comprises Rap2A, Rap2B, and Rap2C and has been implicated in carcinogenesis, regulation of cell adhesion, establishment of cell morphology, and modulation of synapses in neurons [70,74]. GEFs have been reported to have mixed roles in cancer, as they can act either as tumor suppressors or promoters [46]. The role of Rap2 proteins in cancer is also complex. For instance, in hepatocellular carcinoma cells, *Rap2B* has been described as an oncogene, which promotes proliferation and invasion [75]. The *Rap2B* gene has also been described in association with the p53 tumor suppressor involved in cell-cycle arrest, which is a protective mechanism that gives the damaged cell some time to repair the damage [76]. 

Considering the mixed roles of the GEF and Rap proteins in the regulation of oncogenic processes, the role of RASGEF1A in carcinogenesis may also be complex. Currently, the role of RASGEF1A and Rap2 proteins in PBMCs is not known. It is possible that they may be involved in immune responses to tumor, considering that a recent transcriptome analysis of PBMCs revealed a role of the *Ras* genes in host immune responses. More specifically, *K-Ras* and *N-Ras* were downregulated, while *H-Ras* was upregulated in PBMCs of patients with COVID-19 [77]. Interestingly, one study showed that the RASGEF1A protein had GEF activity for K-RAS, H-RAS, and N-RAS proteins in vitro [78], although this finding could not be replicated in a later study [47]. In breast cancer, it is possible that the observed downregulation of the *374459 RASGEF1A* isoform in PBMCs of BC patients may affect the activity of these immune cells. By causing dysregulation of the Rap pathway, it may potentially reduce PBMC activity and contribute to the weakening of antitumor immunity. This would be in line with the observation that *374459* downregulation is most evident in TNBC, which is known as immunologically more evasive among the BC subtypes [79]. However, this postulation requires further experimentation and validation.

### 4.3. RASGEF1A 374459 Isoform and Cancer Proliferation and Shedding

Our results also revealed an association between the decreased *374459* expression and higher Ki-67 and ctDNA values. Ki-67 is a prognostic proliferation marker measured by the clinical pathology labs to assist in treatment decisions. While the margins delineating high and low Ki-67 values vary between laboratories [49], the Ki-67 cutoff point generally depends on the clinical objective: if Ki-67 is used to identify patients sensitive to chemotherapy protocols, it is preferred to set the cut-off at 25% [51,54]. If Ki-67 is used to exclude patients with slowly proliferating tumors from chemotherapeutic protocols, a cut-off of 10% can help avoid overtreatment [51]. Ki-67 is also used in BC classification, with Ki-67 < 14% best correlating with the gene-expression definition of Luminal A [50,52]. The Saint Gallen International Breast Cancer Conference (2011) Expert Panel designated tumors with a Ki-67 < 14% as “low proliferation” [80]. In our study, those BC patients who exhibited the highest Ki-67 values (>50%) had the lowest *374459* expression, suggesting an association between tumor proliferation and the *374459 RASGEF1A* isoform. Interestingly, the Ki-67 values over 50% are most common in TNBC patients [49,53]. 

Additionally, in our study, the patients with lower *374459* expression had higher ctDNA content, as estimated by the mFAST-SeqS genome-wide z-scores. The mFAST-SeqS method detects tumor-specific aneuploidy in circulating cell-free DNA without the requirement for prior knowledge of specific aberrations of the primary tumor. The z-score of 5 predicts a mutant allele frequency (mAF) of 10.5% [56,57]. Overall, our findings that lower *374459* expression correlates with both higher Ki-67 proliferation index as well as ctDNA shedding are in agreement with the studies reporting an association between proliferation and ctDNA [81,82].

### 4.4. Advantages of Isoform-Level Bioinformatics Analysis of RNA-Seq Data

Our isoform-level RNA-seq analysis identified specific BC-associated changes in the *RASGEF1A 374459* transcriptional isoform. It is interesting to note that when an all-inclusive RNA-seq analysis (i.e., including all isoforms for each gene) was performed, the expression of the *RASGEF1A* gene in blood immune cells from our two study groups was not statistically different. However, upon isoform-specific analysis, one of the *RASGEF1A* isoforms was detected as differentially expressed. This suggests that by using the standard RNA-seq bioinformatics analysis, specific isoforms may be missed, which can be disadvantageous in cases where changes in these isoforms are disease-associated and biologically important. Regarding the *RASGEF1A* gene, the *395810* isoform is generally more abundant in blood cells than *374459*, which is a low-expression isoform (Figure 2D). Because of the quantitative preponderance of *395810*, any changes in *374459* expression may fail to be detected in the all-inclusive RNA-seq analysis. Along the same line, biologically relevant gene expression changes could also potentially be missed in other situations, for instance, in the scenario where one of the isoforms of a given gene would be upregulated while another downregulated. Due to the “cancelling out” effect of expression changes (one variant up, one down), the given gene may not be detected as differentially expressed by all-inclusive RNA-seq analysis; the differences would only be observable by isoform analysis. Taking all this into account, it is clear that more specific bioinformatics analyses such as isoform analyses carry particular value in the detection of disease biomarkers.

### 4.5. Other Dysregulated Isoforms Identified in Our Study

Our study of PBMC-expressed isoforms identified two other transcriptional variants that were differentially expressed (i.e., elevated) in TNBC, the *ENST00000589996* variant of the *TBCB* gene and the *ENST00000378603* variant of the *DDB2* gene. TBCB is involved in the folding of β-tubulin and the formation of α/β-tubulin [83], and localizes at spindle and midzone microtubules during mitosis [84]. Abnormal levels of TBCB and TBCE are associated with microtubule abnormalities [85]. Cancer cells are known to depend on their cytoskeleton (including microtubules) to proliferate, invade, and metastasize [86]. TBCB has previously been implicated in cancer [87,88], but only little is known about its involvement in BC. It has been reported that *TBCB* expression is elevated in BC tissues and that *TBCB* overexpression increases the degree of malignancy in BC cell lines [83,89]. 

On the other hand, many studies have linked *DDB2* with cancer [90,91,92,93,94,95]. Due to its role in nucleotide excision repair (NER), it is not surprising that decreased *DDB2* expression has been reported in various cancerous tissues, for instance, in prostate [91], skin [92], head and neck [93], and ovarian cancer [96]. However, DDB2 also has other functions, such as regulation of cell-cycle and transcription, and it appears to have a dual role in cancer. For example, upregulated *DDB2* expression was detected in colorectal cancer tissues [94]. Moreover, introduction of the *DDB2* gene into triple-negative MDA-MB231 cells stimulated growth and colony formation, while *DDB2* knockdown in MCF-7 BC cells caused a decrease in cancer cell growth and colony formation [95]. 

While TBCB and DDB2 do not interact directly (Figure 1B and Figure S1), they are both crucial for maintaining cell cycle integrity—TBCB through its role in mitosis and DDB2 by ensuring genomic stability via DNA damage repair—and are thus functionally linked within broader cellular pathways. Both genomic integrity and proper microtubule dynamics (involved in cell division, intracellular transport, etc.) are essential for preserving the health and functionality of cells, including immune cells such as PBMCs. This, in turn, supports proper immune responses that are vital for preventing transformation toward BC, as disruptions in either process can be deleterious to immune cells and may impair the immune response to tumors.

Our isoform-level RNA-seq analysis highlighted a potentially interesting association between individual BC subtypes and specific *TBCB* and *DDB2* isoforms in PBMCs. No study has yet investigated *TBCB* or *DDB2* isoforms in connection with cancer. Thus, additional investigations are warranted to help unravel the potential utility of the here-identified *TBCB* and *DDB2* isoforms as possible BC biomarkers.

### 4.6. Limitations and Future Perspectives

This case-control study identified a distinctively expressed transcriptional isoform (*374459*) among subject groups. It would be interesting to see how the *374459* and *395810* variants differ at the protein level. Analysis of their protein sequences reveals that they differ in the first exon, with 374459 encoding a protein that is eight amino acids longer at the N-terminus compared to 395810, making these isoforms “N-terminal proteoforms” [97]. While no functional studies have been conducted on RASGEF1A isoforms, making it difficult to determine the exact effect of the N-terminal amino acid difference, our RNA-seq and RT-qPCR results suggest that it is not inconsequential. Importantly, N-terminal proteoforms have been described in human disease [98]. The difference in the 374459 and 395810 isoforms does not affect the RAS-GEF functional domain, which is located away from the N-terminus. However, since UniProt indicates that RASGEF1A is membrane-associated, the additional N-terminal amino acids could potentially influence membrane localization signals [99], affecting isoform localization. Additionally, it is known that N-terminal proteoforms may engage in different protein complexes due to interactions with distinct molecules [97]. The extended N-terminal sequence could also influence the protein’s stability, N-terminal acetylation, folding, and function [97,98,100,101], potentially contributing to distinct properties between the isoforms.

Looking ahead, future mechanistic in vitro studies hold the potential to unveil the functions of RASGEF1A and the 374459 isoform in immune blood cells, which could provide insights into the differential expression in PBMCs of BC patients.

## 5. Conclusions

In conclusion, we performed the first isoform-level transcriptome analysis of PBMCs from breast cancer (BC) patients and identified the *ENST00000374459 RASGEF1A* isoform as BC-associated. The *ENST00000374459 RASGEF1A* isoform levels may have potential as a screening biomarker to differentiate BC patients from healthy subjects. In addition, we found that *ENST00000374459* downregulation in BC was associated with increased Ki67 proliferation index and increased ctDNA shedding. Considering that analyzing the expression of this isoform is less work-intensive and financially more feasible than analyzing ctDNA, this expression analysis could prove useful as a surrogate indicator in the clinical setting for determining disease severity and prognosis. However, this will require further experimental validation.

## Figures and Tables

**Table 1:** Clinicopathological characteristics of BC patients.

Study Cohort: Female Breast Cancer Patients (n = 156)					
BC subtype	Luminal A	Luminal B	HER2(+)	TNBC	
	45 (28.85%)	90 (57.69%)	5 (3.21%)	16 (10.26%)	
Histological type	ILC	IDC	IDC + DCIS	* Other	
	18 (11.54%)	57 (36.54%)	73 (46.79%)	8 (5.13%)	
Localization/containment	Localized/contained	Locally advanced	Metastatic	Not known	
	124 (79.49%)	17 (10.90%)	8 (5.13%)	7 (4.49%)	
Grade	1	2	3	Not known	
	36 (23.08%)	73 (46.79%)	40 (25.64%)	7 (4.49%)	
Tumor size	T1, ≤2 cm	T2, >2 to ≤5 cm	T3, >5 mm	Not known	
	95 (60.90%)	43 (27.56%)	3 (1.92%)	15 (9.61%)	
Lymph nodes	Negative	Micrometastasis ≤ 2 mm	Macrometastasis > 2 mm	Not known	
	98 (62.82%)	7 (4.49%)	23 (14.74%)	28 (17.95%)	
Ki-67 index	<14%	≥14 to ≤25%	>25 to ≤50%	>50 to ≤100%	Not known
	44 (28.21%)	56 (35.90%)	41 (26.28%)	13 (8.33%)	2 (1.28%)
Genome-wide z-score	≤3%	>3%	Not determin.		
	30 (19.23%)	11 (7.05%)	115 (73.72%)		

* Other histological types included mixed, tubular, and cribriform.

**Table 2:** Gene Ontology (GO) for *RASGEF1A*, *DDB2*, and *TBCB* [40].

Gene	GO Term ID	GO Term Name	GO Category	GO Term Description
*RASGEF1A*	GO:0005085	guanyl-nucleotide exchange factor activity	Molecular Function	Stimulates the exchange of GDP to GTP on a signaling GTPase
*RASGEF1A*	GO:0007265	Ras protein signal transduction	Biological Process	Involved in the transmission of signals through Ras proteins
*DDB2*	GO:0003684	Damaged DNA binding	Molecular Function	The ability to bind to DNA that has been damaged
*DDB2*	GO:0006281	DNA repair	Biological Process	Cellular processes of restoring DNA after damage
*TBCB*	GO:0043014	Alpha-tubulin binding	Molecular Function	Binding to the microtubule constituent protein alpha-tubulin
*TBCB*	GO:0007021	Tubulin complex assembly	Biological Process	Assembly of alpha- and beta-tubulin to form a tubulin heterodimer
*TBCB*	GO:0007023	Post-chaperonin tubulin folding pathway	Biological Process	Completion of folding of alpha- and beta-tubulin after chaperonin-mediated partial folding

## Data Availability

RNA-seq data from PBMCs of breast cancer patients were deposited in the NCBI-supported database Gene Expression Ominibus (GEO), accession record GSE270376 https://www.ncbi.nlm.nih.gov/geo/query/acc.cgi?acc=GSE270376; accessed on 13 September 2024.

## Supplementary Materials

The following supporting information can be downloaded at: https://www.mdpi.com/article/10.3390/cancers16183171/s1. Figure S1: STRING interaction networks for RASGEF1A, DDB2, and TBCB; Figure S2: *RASGEF1A 374459* isoform expression and clinicopathological parameters; Table S1: List of annotated isoforms identified as differentially expressed (DEIs) in PBMCs from TNBC compared to luminal A patients.
