# Supplementary material for: Isoform-Level Transcriptome Analysis of Peripheral Blood Mononuclear Cells from Breast Cancer Patients Identifies a Disease-Associated RASGEF1A Isoform
Source: Cancers (Basel). 2024 Sep 16;16(18):3171. doi: 10.3390/cancers16183171 (PMC11429621; doi:10.3390/cancers16183171)
Supplement: Supplementary file 1 [file cancers-16-03171-s001.zip › Figure S1. NEW RASGEF1A.pdf]

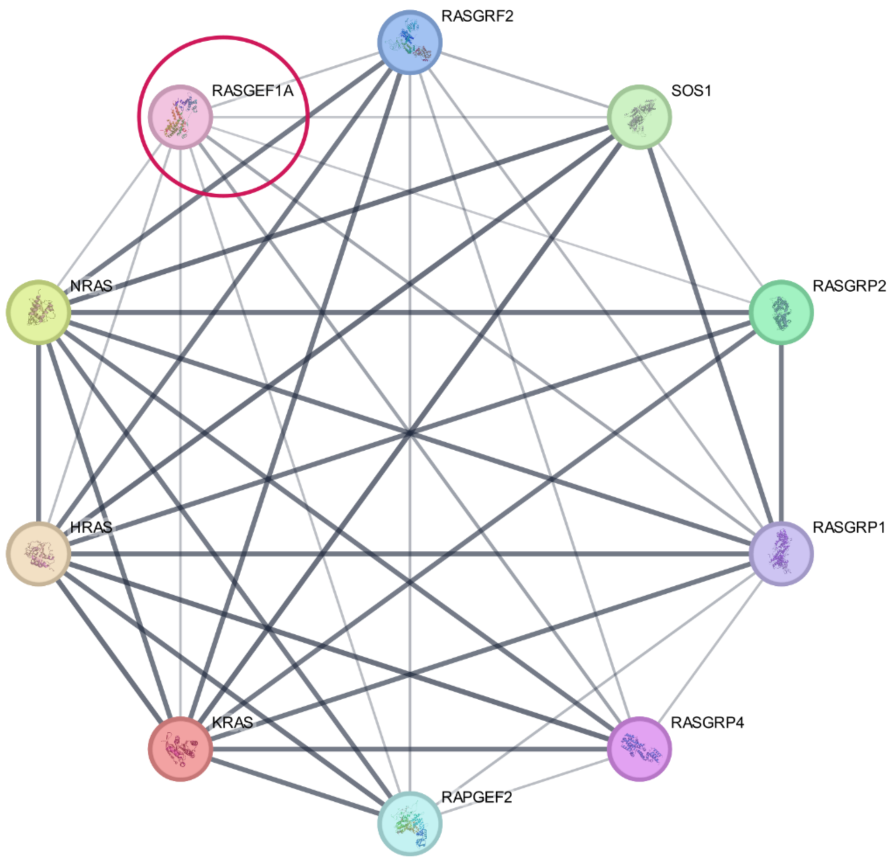
RASGRF2
RASGEF1A
SOS1
NRAS
RASGRP2
HRAS
RASGRP1
KRAS
RASGRP4
RAPGEF2

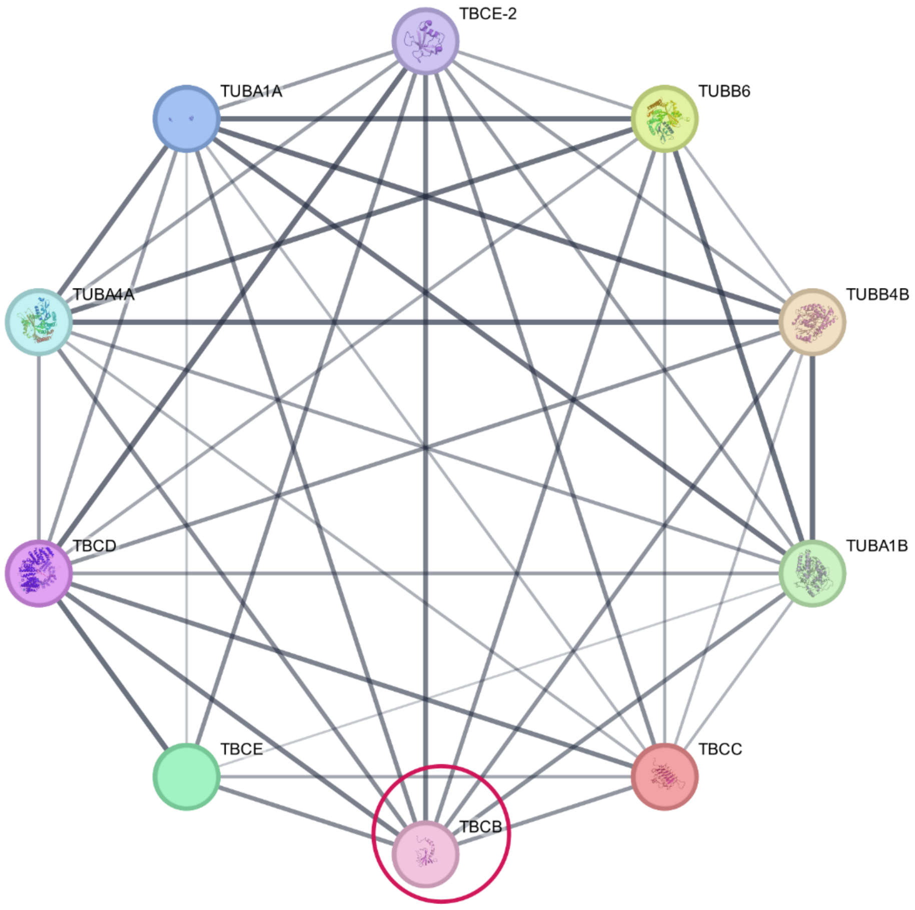
TBCE-2
TUBA1A
TUBB6
TUBA4A
TUBB4B
TBCD
TUBA1B
TBCE
TBCC
TBCB

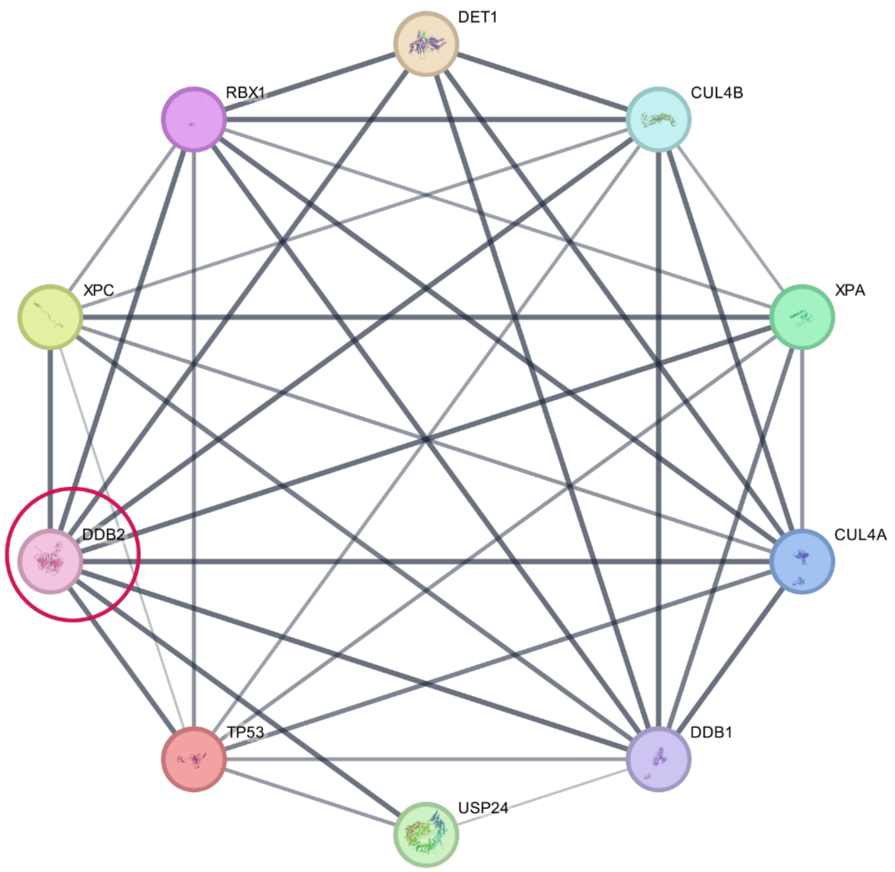


**Figure S1. STRING interaction networks for RASGEF1A, TBCB and DDB2**. The STRING database was used to construct a protein–protein interaction network. Line thickness indicates the strength of data support.
