# Supplementary material for: Isoform-Level Transcriptome Analysis of Peripheral Blood Mononuclear Cells from Breast Cancer Patients Identifies a Disease-Associated RASGEF1A Isoform
Source: Cancers (Basel). 2024 Sep 16;16(18):3171. doi: 10.3390/cancers16183171 (PMC11429621; doi:10.3390/cancers16183171)
Supplement: Supplementary file 1 [file cancers-16-03171-s001.zip › Figure S2. RASGEF1A.pdf]

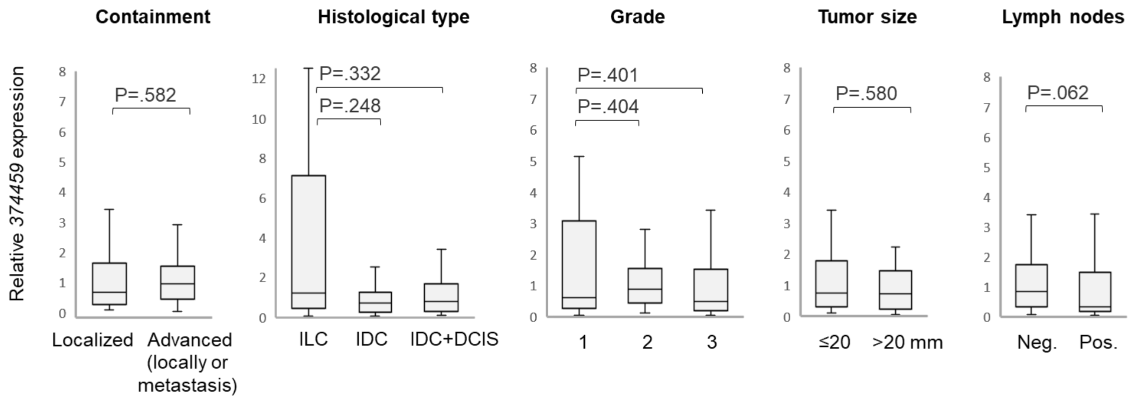

Figure S2. *RASGEF1A 374459* isoform expression and clinicopathological parameters. Expression analysis of *374459* in PBMCs from BC patients stratified by (i) cancer containment (localized vs. advanced locally or metastasis), (ii) histological type (ILC: invasive lobular carcinoma, IDC: invasive ductal carcinoma, DCIS: ductal carcinoma in situ), (iii) grade, (iv) tumour size, and (v) lymph node invasion. Mann-Whitney U test, *p<.05.
